# Promoter Recognition by a Complex of Spx and the C-Terminal Domain of the RNA Polymerase α Subunit

**DOI:** 10.1371/journal.pone.0008664

**Published:** 2010-01-13

**Authors:** Michiko M. Nakano, Ann Lin, Cole S. Zuber, Kate J. Newberry, Richard G. Brennan, Peter Zuber

**Affiliations:** 1 Department of Science & Engineering, School of Medicine, Oregon Health & Science University, Beaverton, Oregon, United States of America; 2 Department of Biochemistry and Molecular Biology, University of Texas, M. D. Anderson Cancer Center, Texas, United States of America; University of Liverpool, United Kingdom

## Abstract

**Background:**

Spx, an ArsC (arsenate reductase) family member, is a global transcriptional regulator of the microbial stress response and is highly conserved amongst Gram-positive bacteria. *Bacillus subtilis* Spx protein exerts positive and negative control of transcription through its interaction with the C-terminal domain of the RNA polymerase (RNAP) α subunit (αCTD). Spx activates *trxA* (thioredoxin) and *trxB* (thioredoxin reductase) in response to thiol stress, and bears an N-terminal C10XXC13 redox disulfide center that is oxidized in active Spx.

**Methodology/Principal Findings:**

The structure of mutant Spx^C10S^ showed a change in the conformation of helix α4. Amino acid substitutions R60E and K62E within and adjacent to helix α4 conferred defects in Spx-activated transcription but not Spx-dependent repression. Electrophoretic mobility-shift assays showed αCTD interaction with *trxB* promoter DNA, but addition of Spx generated a supershifted complex that was disrupted in the presence of reductant (DTT). Interaction of αCTD/Spx complex with promoter DNA required the *cis*-acting elements -45AGCA-42 and -34AGCG-31 of the *trxB* promoter. The Spx^G52R^ mutant, defective in αCTD binding, did not interact with the αCTD-*trxB* complex. Spx^R60E^ not only failed to complex with αCTD-*trxB*, but also disrupted αCTD-*trxB* DNA interaction.

**Conclusions/Significance:**

The results show that Spx and αCTD form a complex that recognizes the promoter DNA of an Spx-controlled gene. A conformational change during oxidation of Spx to the disulfide form likely alters the structure of Spx α helix α4, which contains residues that function in transcriptional activation and αCTD/Spx-promoter interaction. The results suggest that one of these residues, R60 of the α4 region of oxidized Spx, functions in αCTD/Spx-promoter contact but not in αCTD interaction.

## Introduction

Spx is a member of the ArsC (arsenate reductase) family of proteins and is a unique transcriptional regulator of Gram-positive bacteria [1]. In *Bacillus subtilis*, its primary function is in the global control of the thiol stress response [2]. A role for Spx in pathogenesis has been inferred from studies of *Staphylococcus aureus*, *Listeria monocytogenes*, *Bacillus anthracis*, and *Streptococcus mutans*, in which it is produced upon host cell infection and serves to control the expression of virulence determinants [3], [4], [5], [6], [7]. *B. subtilis* Spx interacts with the C-terminal domain of the α subunit (αCTD) of RNA polymerase (RNAP), and in doing so, exerts both positive and negative control of gene transcription [2], [8]. Genetic and biochemical studies showed that the G52 residue of Spx and the Y263 residue of αCTD form part of the contact interface between Spx and RNAP, which was confirmed by the x-ray structure determination of the αCTD/Spx complex [9]. The Y263 is conserved in Gram-positive bacteria and is not found in the Gram-negatives [1].

In vitro transcription experiments showed that Spx directly activates transcription of genes involved in thiol homeostasis, including *trxA* (thioredoxin gene) and *trxB* (thioredoxin reductase) [10], [11]. Spx-dependent transcriptional activation is under redox control, requiring a CXXC disulfide center at the protein's N-terminus [10]. The oxidized form of Spx activates transcription of genes under its control.

Upon oxidative stress, Spx concentrations increase due to enhanced transcription and decreased proteolysis. Increased *spx* transcription is attributed, in part, to inactivation of the PerR and YodR repressors that negatively control *spx* transcription during non-stress conditions [12], [13]. Spx is also under proteolytic control requiring the ATP-dependent ClpXP protease [8], [14], but Spx is able to escape from the proteolysis under oxidative conditions because both the ClpX ATPase subunit and YjbH, an Spx-specific adaptor protein involved in ClpXP proteolysis of Spx, are inactivated by oxidants [15], [16], [17]. YjbH, a Zn-binding protein, interacts with Spx and the interaction accelerates the proteolysis of Spx by ClpXP [15]. Oxidative stress releases Zn from YjbH and ClpX, which is thought to result in loss of YjbH-mediated proteolysis.

The tight transcriptional and post-translational control of intracellular Spx is logical from a physiological viewpoint, given that the Spx-dependent regulation is exerted over a genome-wide scale. When Spx levels increase, as a result of either a null mutation in *clpX* or *clpP*, or in response to oxidative stress, transcription of a large set of genes is reduced [2], [8]. Most, if not all, of these genes are positively controlled by transcription activators. For example, ComA-dependent *srfA* transcription is repressed when Spx is overproduced. ComA binds to the regulatory region of *srfA* and recruits RNAP to the promoter region through the interaction of ComA and αCTD [18], [19], [20]. ComA-dependent *srfA* transcription requires residues C265 and K267 located in the α1 helix of αCTD [20]. Y263, the residue of αCTD that interacts with Spx, is also located in the α1 helix, indicating that Spx likely competes for binding of αCTD with ComA [9], [20]. The adverse effect of Spx on ResD-dependent transcription of *fnr* was explained by a similar interference of ResD-αCTD interaction by Spx [21].

The interference model of activator-αCTD complex disruption by Spx is consistent with the observation that free Spx does not bind to DNA [10]. Furthermore, the DNA sequence-independent mechanism of action can explain why Spx adversely affects transcription of a wide range of genes. On the other hand, it remains unresolved whether Spx-dependent transcriptional activation involves binding of Spx to a specific sequence within target promoter DNA. A previous study showed that oxidized Spx alone does not bind to the *trxA* or *trxB* promoters [10], although this does not necessarily eliminate the possibility that Spx, when complexed with RNAP, directly binds to a sequence within an Spx-activated promoter. In fact, previous DNase I footprinting analyses showed that neither RNAP nor Spx individually binds to *trxA* and *trxB*, while the presence of both RNAP and Spx resulted in footprints covering a region between −50 and −10 within each promoter [10]. This result indicates that Spx, after forming a complex with αCTD, directs RNAP to Spx-activated promoters, either by altering the way in which RNAP engages the promoter, or by forming part of the binding surface which specifically recognizes Spx-activated promoter sequences. In the hope of obtaining direct evidence of a DNA-protein interaction in the transcription initiation complex at the *trxA* and *trxB* promoters, we carried out site-specific DNA and protein crosslinking using *trxA* (and *trxB*) promoter DNA, RNAP, and Spx [11]. Contact of Spx with promoter DNA was not detected by crosslinking at any of the nucleotide positions examined. The addition of Spx resulted in enhanced σ^A^ contact with the −10 region of the *trxA* and *trxB* promoter at 37°C. Similarly, Spx stimulated contact of the ββ' subunits of RNAP with nucleotide base positions near the transcription start sites and around −21/−22 in both promoters. That study also uncovered evidence of a *cis*-acting element upstream of the core promoter sequences in Spx-controlled genes that is required for Spx-activated transcription [11].

In this work, we identify the *cis*-sequence in the *trxB* promoter that is essential for Spx-dependent transcriptional activation. As previously shown, Spx alone is unable to bind the *trxB* promoter, but Spx is capable of generating a supershifted *trxB*-αCTD-Spx complex in an electrophoretic mobility shift assay (EMSA). In parallel, structural studies identify a change in the conformation of a helix α4 of Spx when residue C10 of the redox disulfide center at its N-terminus is mutated to serine. When R60, a residue associated with the helix α4 region, is mutated to glutamate, Spx-dependent transcription of *trxB in vivo* and binding of the αCTD-Spx complex to the *trxB* promoter *in vitro* are abolished.

## Materials and Methods

### Crystallization and Structural Analysis of Reduced Spx

“Reduced” Spx, in which residue Cys10 was replaced by serine (C10S Spx), was overexpressed in *E. coli* and purified by Ni^2+^-NTA affinity column chromatography as previously described [9]. Purified, cleaved Spx was dialyzed into 25 mM PIPES, pH 6.5, 100 mM KCl for use in subsequent crystallization trials.

αCTD containing residues 245 to 314 of RpoA was cloned into the IPTG-inducible PET28a overexpression vector (Invitrogen), which contains an N-terminal histidine tag and thrombin cleavage site. The αCTD-PET28a vector was transformed into Rosetta 2 competent cells (EMD Biosciences) for overexpression. αCTD was purified by Ni-NTA affinity chromatography (Qiagen). Following purification, the histidine tag was cleaved by incubation with thrombin protease (GE Healthsciences). Purified, cleaved αCTD was dialyzed into 25 mM Pipes, pH 6.5, 100 mM KCl. The Spx-αCTD complex was formed by mixing equimolar amounts of purified C10S Spx and αCTD. The complex was concentrated to 10 mg/ml.

Crystals of the reduced C10S Spx-αCTD complex were grown by the hanging-drop vapor diffusion method. Two microliters of 0.5 mM C10S Spx-αCTD was mixed with 2 µL of reservoir solution containing 25%–30% polyethylene glycol 4000, 0.1 M sodium citrate pH 5.3 and 0.1 M MgCl_2_. Crystals appeared within two to three days and reached maximum dimensions of 0.3×0.05×0.05 mm^3^ in approximately one week. The crystals were cryoprotected in the mother liquor plus the addition of 15% glycerol and flash frozen in a nitrogen stream at −180°C. X-ray intensity data were collected on beamline 8.3.1 at the Advanced Light Source and data were processed using MOSFLM [22] as implemented in the CCP4 suite of programs (CCP4, 2004) (Table 1). The crystals took the spacegroup P1 with cell dimensions a = 29.4 Å, b = 32.2 Å, c = 50.6 Å, α = 106.1°, β = 91.6°, γ = 103.8°.

**Table 1:** Selected Crystallographic Data and Statistics.

	SpxC10S-αCTD
**Data Collection**	
Wavelength (Å)	1.0000
Resolution (Å)	48.3–1.9
No. Observed Reflections	174,032
No. Unique Reflections	12,912
Completeness (%) (last shell)	95.1 (94.9)
I/σI (last shell)	7.7 (2.9)
R_sym_ (%)^a^ (last shell)	5.9 (23.0)
**Refinement**	
Resolution (Å)	48.3–1.9
Reflections (working set/test set)	12,250/662
Protein Atoms (#)	1,465
Solvent Molecules (#)	40
R_work_/R_free_ (%)^b^	23.6/27.8
RMSD bond lengths (Å)	0.006
RMSD bond angles (°)	1.27
Average B-factor (Å^2^)	38.5

a R_sym_ = ∑∑|Ihkl-Ihkl(j)|/∑Ihkl, where Ihkl(j) is the observed intensity and Ihkl is the final average value of intensity.

b R_work_ = ∑||F_obs_|−|F_calc_||/∑|F_obs_| and R_free_ = ∑||F_obs_|−|F_calc_||/∑|F_obs_|; where all reflections belong to a test set of 5% randomly selected data.

The structure of the C10S Spx-αCTD complex was solved by molecular replacement using the oxidized Spx-αCTD structure as the search model (Table 1). Manual fitting and adjustment of the model resulted in the placement of amino acid residues 1–115 of Spx and residues 245–311 of αCTD into the electron density map using O [23]. The initial protein model was subjected to rigid body refinement, followed by simulated annealing and positional and B-factor refinement using CNS [24]. Simulated-annealing omit maps were calculated to ensure the correct placement of all residues and to avoid model bias. The final model included residues 1–115 of Spx, residues 245–311 of αCTD, and 40 solvent molecules. The final R_work_ and R_free_ are 23.6% and 27.8%, respectively, to 1.9 Å resolution. The stereochemistry of the final model was assessed with PROCHECK [25], which revealed 89.7% of all φ/ψ angles in the most favored regions of the Ramachandran plot and none in the disallowed regions. The coordinates and structure factors have been deposited in the RCSB with accession number 3IHQ.

### Construction of Spx Amino Acid Substitution Mutants

All bacterial strains and plasmids are listed in Table 2. The effect of Spx amino acid substitution was examined with the Spx construct carrying amino acid substitutions (AN to DD) at the carboxyl-terminal end, which renders Spx insensitive to ClpXP proteolysis [2]. The previously constructed plasmids pSN56 [2] and pZY14 [20] are pDR111 derivatives that carry *spx^DD^* and *spx^C10A-DD^*, respectively. pDR111 is an *amyE* integration vector, and the cloned *spx* genes are transcribed from the IPTG-inducible P*spankhy* promoter [26].

**Table 2:** *B. subtilis* strains and plasmids.

Strain or plasmid	Relevant genotype or characteristics	
***B. subtilis*** ** strains**		
JH642	parental strain	James Hoch
ORB3834	Δ*spx*::*neo*	[31]
ORB4028	Δ*spx*::*neo his_10_-rpoC*	[11]
ORB4055	*spx^G52R^*	[31]
ORB4566	Δ*spx*::*neo thrC*::*trxB*(−510 to +190)*-lacZ*	[10]
ORB6129	*amyE*::P*spankhy-spx^DD^ srfA-lacZ*	[20]
ORB6894	Δ*spx*::*neo thrC*::*trxB*(−510 to +190)*-lacZ amyE*::P*spankhy-spx^DD^*	This study
ORB6895	Δ*spx*::*neo thrC*::*trxB*(−510 to +190)*-lacZ amyE*::P*spankhy-spx^DD-R60E^*	This study
ORB6896	Δ*spx*::*neo thrC*::*trxB*(−510 to +190)*-lacZ amyE*::P*spankhy-spx^DD-K62E^*	This study
ORB6897	Δ*spx*::*neo thrC*::*trxB*(−510 to +190)*-lacZ amyE*::P*spankhy-spx^DD-K66E^*	This study
ORB6930	*amyE*::P*spankhy-spx^DD-R60E^*	This study
ORB6931	*amyE*::P*spankhy-spx^DD-K62E^*	This study
ORB6932	*amyE*::P*spankhy-spx^DD-K66E^*	This study
ORB6934	*amyE*::P*spankhy-spx^DD-R60E^ srfA-lacZ*	This study
ORB6935	*amyE*::P*spankhy-spx^DD-K62E^ srfA-lacZ*	This study
ORB6936	*amyE*::P*spankhy-spx^DD-K66E^ srfA-lacZ*	This study
ORB7271	Δ*spx*::*neo thrC*::*trxB*(G-44C)*-lacZ amyE*::P*spankhy-spx^DD^*	This study
ORB7272	Δ*spx*::*neo thrC*::*trxB*(G-44A)*-lacZ amyE*::P*spankhy-spx^DD^*	This study
ORB7273	Δ*spx*::*neo thrC*::*trxB*(G-33A)*-lacZ amyE*::P*spankhy-spx^DD^*	This study
ORB7274	Δ*spx*::*neo thrC*::*trxB*(G-31A)*-lacZ amyE*::P*spankhy-spx^DD^*	This study
ORB7275	Δ*spx*::*neo thrC*::*trxB*(G-44T)*-lacZ amyE*::P*spankhy-spx^DD^*	This study
ORB7276	Δ*spx*::*neo thrC*::*trxB-lacZ amyE*::P*spankhy-spx^DD^*	This study
ORB7282	Δ*spx*::*neo thrC*::*trxB-lacZ amyE*::P*spankhy-spx^DD-R60E^*	This study
ORB7316	Δ*spx*::*neo thrC*::*trxB-lacZ amyE*::P*spankhy-spx^DD-C10A^*	This study
ORB7337	Δ*spx*::*neo thrC*::*trxB-lacZ amyE*::P*spankhy-spx^DD-G52R^*	This study
ORB7322	Δ*spx*::*neo thrC*::*trxB*(C-32A)*-lacZ amyE*::P*spankhy-spx^DD^*	This study
ORB7342	Δ*spx*::*neo thrC*::*trxB*(A-34T)*-lacZ amyE*::P*spankhy-spx^DD^*	This study
ORB7343	Δ*spx*::*neo thrC*::*trxB*(A-34T)*-lacZ amyE*::P*spankhy-spx^DD-R60E^*	This study
ORB7347	Δ*spx*::*neo thrC*::*trxB*(A-34T)*-lacZ amyE*::P*spankhy-spx^DD-C10A^*	This study
ORB7348	Δ*spx*::*neo thrC*::*trxB*(A-34T)*-lacZ amyE*::P*spankhy-spx^DD-G52R^*	This study
ORB7349	Δ*spx*::*neo thrC*::*trxB*(C-32T)*-lacZ amyE*::P*spankhy-spx^DD^*	This study
ORB7356	Δ*spx*::*neo thrC*::*trxB*(G-31C)*-lacZ amyE*::P*spankhy-spx^DD^*	This study
ORB7368	Δ*spx*::*neo thrC*::*trxB*(A-45T)*-lacZ amyE*::P*spankhy-spx^DD^*	This study
ORB7375	Δ*spx*::*neo thrC*::*trxB*(A-42T)*-lacZ amyE*::P*spankhy-spx^DD^*	This study
ORB7426	Δ*spx*::*neo thrC*::*trxB*(G-31T)*-lacZ amyE*::P*spankhy-spx^DD^*	This study
ORB7427	Δ*spx*::*neo thrC*::*trxB*(A-42G)*-lacZ amyE*::P*spankhy-spx^DD^*	This study
ORB7488	Δ*spx*::*neo thrC*::*trxB*(C-43G)*-lacZ amyE*::P*spankhy-spx^DD^*	This study
ORB7491	Δ*spx*::*neo thrC*::*trxB*(C-43T)*-lacZ amyE*::P*spankhy-spx^DD^*	This study
ORB7540	Δ*spx*::*neo thrC*::*trxB*(A-34G)*-lacZ amyE*::P*spankhy-spx^DD^*	This study
ORB7541	Δ*spx*::*neo thrC*::*trxB*(A-34C)*-lacZ amyE*::P*spankhy-spx^DD^*	This study
ORB7592	Δ*spx*::*neo thrC*::*trxB*(A-37C)*-lacZ amyE*::P*spankhy-spx^DD^*	This study
ORB7593	Δ*spx*::*neo thrC*::*trxB*(A-39C)*-lacZ amyE*::P*spankhy-spx^DD^*	This study
ORB7594	Δ*spx*::*neo thrC*::*trxB*(C-43A)*-lacZ amyE*::P*spankhy-spx^DD^*	This study
ORB7595	Δ*spx*::*neo thrC*::*trxB*(T-47G)*-lacZ amyE*::P*spankhy-spx^DD^*	This study
ORB7596	Δ*spx*::*neo thrC*::*trxB*(G-49T)*-lacZ amyE*::P*spankhy-spx^DD^*	This study
**Plasmid**		
pCSZ28	pDG793 with *trxB*(G-31T)-*lacZ*	This study
pCSZ29	pDG793 with *trxB*(A-42G)-*lacZ*	This study
pDG793	*thrC* integration vector with promoter-less *lacZ*	[28]
pDYR9	pDG793 with *trxB-lacZ*	[11]
pDYR24	pDG793 with *trxB*(A-37C)-*lacZ*	[11]
pDYR25	pDG793 with *trxB*(A-39C)-*lacZ*	[11]
pDYR26	pDG793 with *trxB*(C-43A)-*lacZ*	[11]
pDYR27	pDG793 with *trxB*(G-44T)-*lacZ*	[11]
pDYR28	pDG793 with *trxB*(T-47G)-*lacZ*	[11]
pDYR29	pDG793 with *trxB*(G-49T)-*lacZ*	[11]
pDR111	*amyE* integration vector with P*spankhy* promoter	[26]
pMMN92	SPβ carrying *srfA-lacZ*	[18]
pMMN470	pTYB4 with *spx*	[31]
pMMN683	pDR111 with *spx^DD-R60E^*	This study
pMMN684	pDR111 with *spx^DD-k62E^*	This study
pMMN685	pDR111 with *spx^DD-K66E^*	This study
pMMN738	pTYB4 with *spx^R60E^*	This study
pMMN745	pDG793 with *trxB*(G-44C)-*lacZ*	This study
pMMN746	pDG793 with *trxB*(G-44A)-*lacZ*	This study
pMMN747	pDG793 with *trxB*(G-33A)-*lacZ*	This study
pMMN748	pDG793 with *trxB*(G-31A)-*lacZ*	This study
pMMN752	pDG793 with *trxB*(C-32A)-*lacZ*	This study
pMMN753	pUC19 with *spx^DD-G52R^*	This study
pMMN754	pDR111 with *spx^DD-G52R^*	This study
pMMN755	pDG793 with *trxB*(A-34T)-*lacZ*	This study
pMMN756	pDG793 with *trxB*(C-32T)-*lacZ*	This study
pMMN757	pDG793 with *trxB*(G-31C)-*lacZ*	This study
pMMN758	pDG793 with *trxB*(A-45T)-*lacZ*	This study
pMMN759	pDG793 with *trxB*(A-42T)-*lacZ*	This study
pMMN763	pDG793 with *trxB*(C-43G)-*lacZ*	This study
pMMN764	pDG793 with *trxB*(C-43T)-*lacZ*	This study
pMMN775	pDG793 with *trxB*(A-34G)-*lacZ*	This study
pMMN776	pDG793 with *trxB*(A-34C)-*lacZ*	This study
pSN21	pTYB4 with *spx^G52R^*	[8]
pSN37	pTYB2 with *rpoA*-CTD	[8]
pSN56	pDR111 with with *spx^DD^*	[2]
pSN64	pTYB4 with *sigA*	[34]
pTYB4	protein expression vector with intein tag	New England Biolabs
pUC19	cloning vector	
pZY14	pDR111 with *spx^DD-C10A^*	[17]

Unless otherwise noted, *trxB-lacZ* contains a region between -115 and +47 of the *trxB* promoter.

Three additional *spx* mutations conferring single amino acid substitutions were generated by two-step PCR-based mutagenesis using a pair of complementary mutagenic oligonucleotides − oMMN07-351 and oMMN07-352 for R60E, oMMN07-353 and oMMN07-354 for K62E, and oMMN07-355 and oMMN07-356 for K66E. Each oligonucleotide pair was used for the first PCR, together with either the upstream oligonucleotide oMMN01-173 or the downstream oligonucleotide oMMN01-174 and the plasmid pSN56 as template. The two PCR products carrying short complementary ends were annealed, filled-in by ExTaq polymerase (Takara Bio USA), and used as template for the second-round of PCR using oMMN01-173 and oMMN01-174. The PCR product was digested with SalI and HindIII and cloned into pDR111 and digested with the same enzymes to generate pMMN683 (*spx^DD-R60E^*), pMMN684 (*spx^DD-K62E^*), and pMMN685 (*spx^DD-K66E^*).

Plasmid pMMN754 carrying *spx^DD-G52R^* was generated as follows. The *spx* gene carrying the G52R mutation was amplified from chromosomal DNA isolated from ORB4055 using oligonucleotides oMMN01-173 and oMMN01-174. The PCR product was digested with HindIII and BclI and the 5′-end of *spx* containing the G52R mutation was isolated. The 3′-end of *spx* carrying the DD mutation was isolated from pSN56 digested with BclI and SalI. The two fragments were cloned into pUC19 digested with HindIII and SalI by three-fragment ligation to generate pMMN753. The *spx* fragment was isolated from pMMN753 digested with HindIII and SalI and cloned into the HindIII-SalI sites of pDR111 to generate pMMN754.

The effect of the R60E, K62E, and K66E mutations of Spx on *trxB* expression was determined by measuring *lacZ* expression driven by the *trxB* promoter (−510 to +190 relative to the transcription start site) as previously described [10]. Plasmids pMMN683 (*spx^DD-R60E^*), pMMN684 (*spx^DD-K62E^*), and pMMN685 (*spx^DD-K66E^*) were used to transform ORB4566 carrying *spx*::*neo* and *trxB-lacZ* at *thrC*, and transformants were selected for spectinomycin resistance (Spc^r^) to generate ORB6895, ORB6896, and ORB6897, respectively. The transformants were screened for the amylase-negative phenotype, which is indicative of double-crossover recombination [27]. As a control, pSN56 carrying the wild-type Spx^DD^ was used to transform ORB4566, and ORB6894 was obtained.

The strains carrying *srfA-lacZ* and the wild-type or mutant Spx^DD^ were constructed as follows. The strain JH642 was transformed with pMMN683, pMMN684, and pMMN685, and the strains ORB6930, ORB6931 and ORB6932 were constructed as described above. Each strain was transduced with SPβ phage carrying pMMN92-borne *srfA-lacZ* [18] to generate ORB6934, ORB6935, and ORB6936. A control strain, ORB6129, carrying the wild-type Spx^DD^ at the *thrC* locus and the *srfA-lacZ* fusion was constructed as previously described [20].

### Construction of *trxB* Promoter Mutations

All mutant *trxB* promoters are derivatives of pDYR9 [11], which carries the *trxB* promoter (−115 to +47) fused to *lacZ*. Base substitution mutations of the *trxB* promoter were constructed by two-step PCR using complementary mutagenic primer pairs in a procedure similar to that used for the amino acid substitutions of Spx. The sequences of mutagenic oligonucleotides are listed in Table 3, and the outside forward (oDYR07-52) and reverse primers (oDYR07-32) were previously described [11]. DNA fragments resulting from the second PCR were digested with EcoRI and HindIII and cloned into pDG793 [28] that had been digested with the same enzymes. pDG793 is a *thrC* integration plasmid and double-crossover recombination is selected by Thr^-^ phenotype. Each plasmid was used to transform ORB3834 (*spx*::*neo*), and erythromycin-resistant (Erm^r^) Thr^-^ transformants were then transformed with chromosomal DNA isolated from strains carrying the wild-type and mutant *spx^DD^* at the *amyE* locus. All plasmids are listed in Table 2.

**Table 3:** Oligonucleotides used in this study.

Oligonucleotide	Sequence	Purpose
oMMN01-135	AGAGGAGTGAAGATCCATGGTTACACTATAC	*spx* forward
oMMN01-137	TAACTCCCGGGGTTTGCCAAACGCTGTGCTT	*spx* reverse
oMMN07-351	GAAATCATCTCAACCGAGTCAAAAGTATTCCAA	*spx* R60E forward
oMMN07-352	TTGGAATACTTTTGACTCGGTTGAGATGATTTC	*spx* R60E reverse
oMMN07-353	ATCTCAACCCGTTCAGAAGTATTCCAAAAACTG	*spx* K62E forward
oMMN07-354	CAGTTTTTGGAATACTTCTGAACGGGTTGAGAT	*spx* K62E reverse
oMMN07-355	TCAAAAGTATTCCAAGAACTGAATGTGAACGTT	*spx* K66E forward
oMMN07-356	AACGTTCACATTCAGTTCTTGGAATACTTTTGA	*spx* K66E reverse
oMMN08-405	ATCGTGTTGAACAAAAAAATAGCGTATC	*trxB* G-44A forward
oMMN08-406	GATACGCTATTTTTTTGTTCAACACGAT	*trxB* G-44A reverse
oMMN08-407	ATCGTGTTGACCAAAAAAATAGCGTATC	*trxB* G-44C forward
oMMN08-408	GATACGCTATTTTTTTGGTCAACACGAT	*trxB* G-44C reverse
oMMN08-409	GAGCAAAAAAATAACGTATCACCATGAGA	*trxB* G-33A forward
oMMN08-410	TCTCATGGTGATACGTTATTTTTTTGCTC	*trxB* G-33A reverse
oMMN08-411	GAGCAAAAAAATAGCATATCACCATGAGA	*trxB* G-31A forward
oMMN08-412	TCTCATGGTGATATGCTATTTTTTTGCTC	*trxB* G-31A reverse
oMMN08-413	GAGCAAAAAAATAGAGTATCACCATGAGA	*trxB* C-32A forward
oMMN08-414	TCTCATGGTGATACTCTATTTTTTTGCTC	*trxB* C-32A reverse
oMMN08-415	GAGCAAAAAAATTGCGTATCACCATGAGA	*trxB* A-34T forward
oMMN08-416	TCTCATGGTGATACGCAATTTTTTTGCTC	*trxB* A-34T reverse
oMMN08-417	GAGCAAAAAAATAGTGTATCACCATGAGA	*trxB* C-32T forward
oMMN08-418	TCTCATGGTGATACACTATTTTTTTGCTC	*trxB* C-32T reverse
oMMN08-419	GAGCAAAAAAATAGCCTATCACCATGAGA	*trxB* G-31C forward
oMMN08-420	TCTCATGGTGATAGGCTATTTTTTTGCTC	*trxB* G-31C reverse
oMMN08-421	TTTAATCGTGTTGTGCAAAAAAATAGCG	*trxB* A-45T forward
oMMN08-422	CGCTATTTTTTTGCACAACACGATTAAA	*trxB* A-45T reverse
oMMN08-423	TAATCGTGTTGAGCTAAAAAATAGCGTA	*trxB* A-42T forward
oMMN08-424	TACGCTATTTTTTAGCTCAACACGATTA	*trxB* A-42T reverse
oMMN08-436	GAGCAAAAAAATAGCTTATCACCATGAGA	*trxB* G-31T forward
oMMN08-437	TCTCATGGTGATAAGCTATTTTTTTGCTC	*trxB* G-31T reverse
oMMN08-438	TAATCGTGTTGAGCGAAAAAATAGCGTA	*trxB* A-42G forward
oMMN08-439	TACGCATTTTTTCGCTCAACACGATTA	*trxB* A-42G reverse
oMMN08-440	AATCGTGTTGAGGAAAAAAATAG	*trxB* C-43G forward
oMMN08-441	CTATTTTTTTCCTCAACACGATT	*trxB* C-43G reverse
oMMN08-442	AATCGTGTTGAGTAAAAAAATAG	*trxB* C-43T forward
oMMN08-443	CTATTTTTTTACTCAACACCGATT	*trxB* C-43T reverse
oMMN08-461	GAGCAAAAAAATGGCGTATCACCATGAGA	*trxB* A-34G forward
oMMN08-462	TCTCATGGTGATACGCCATTTTTTTGCTC	*trxB* A-34G reverse
oMMN08-463	GAGCAAAAAAATCGCGTATCACCATGAGA	*trxB* A-34C forward
oMMN08-464	TCTCATGGTGATACGCGATTTTTTTGCTC	*trxB* A-34C reverse
oMMN08-465	TAATCGTGTTGAGCAAAAAAATAGCGTATCACCATG	*trxB* (−56 to -21)
oMMN08-466	CATGGTGATACGCTATTTTTTTGCTCAACACGATT	*trxB* (−21 to -55)
oMMN08-473	TAATCGTGTTGAACAAAAAAATAACGTATCACCATG	*trxB* G-44A G-33A forward
oMMN08-474	CATGGTGATACGTTATTTTTTTGTTCAACACGATT	*trxB* G-44A G-33A reverse

### Measurement of β-galactosidase Activity

The effect of the Spx amino acid substitutions on the expression of *trxB* and *srfA* was determined by measuring β-galactosidase activity in cells carrying *trxB-lacZ* (ORB6894 to ORB6897) and *srfA-lacZ* (ORB6129, ORB6930 to ORB6932) in the presence and absence of IPTG. The strains were grown at 37°C overnight on DS agar plates [29] supplemented with spectinomycin and erythromycin (for *trxB-lacZ*) or spectinomycin and chloramphenicol (for *srfA-lacZ*). The overnight cultures were used to inoculate the same liquid medium at a starting optical density of 600 nm (OD_600_) of 0.02. When the OD_600_ of the cultures reached 0.4 to 0.5, the cultures were divided into two flasks and 1 mM IPTG was added to one of the flasks. Samples were taken at 0.5- to 1-hr intervals to assay β-galactosidase activity, which was expressed as Miller units [30].

### Western Blot Analysis

The strains ORB6894 (Spx^DD^), ORB6895 (Spx^DD-R60E^), ORB6896 (Spx^DD-K62E^), and ORB6897 (Spx^DD-K66E^) were cultured in DS liquid medium supplemented with spectinomycin and erythromycin as described above. Each culture was divided into two tubes at an OD_600_ of 0.4 to 0.5, and 1 mM IPTG was added to one of the tubes. Two milliliter samples were harvested after a 1.5-hr incubation and resuspended with 0.5 ml of 20 mM potassium phosphate buffer pH 7.5, 15 mM MgCl_2_, 20% sucrose. Lysozyme (1 mg/ml) was added and the suspension was incubated by gently shaking at 37°C for 30 min. The protoplasts were collected by centrifugation at 7,000 x g for 5 min and washed once with the same buffer. The precipitated protoplasts were lysed by resuspending with 0.5 ml of lysis buffer (30 mM Tris-HCl, pH 8.0, 1 mM EDTA) to obtain crude extract. Protein concentrations in the crude extract were determined using BioRad protein assay solution, and 15 µg of total protein was applied to an SDS-polyacrylamide (15%) gel. The Western blot experiment was done as previously described using an anti-Spx antibody [31].

### Protein Purification

RNAP was purified from ORB4028 (*spx*::*neo his_10_-rpoC*) as previously described using a Ni-NTA affinity column, a heparin agarose, and a Bio-Rad High Q column [11], [32], [33]. The self-cleavable intein system (New England Biolabs) was used for overproduction and purification of αCTD and Spx. αCTD (residues 225–314) was overproduced in *Escherichia coli* BL21/pLysS carrying pSN37 [8] and purified using a chitin column and a BioRad High Q column. σ^A^ was overproduced using pSN64 in *E. coli* ER2566 (New England Biolabs) and purified using a chitin column and a High Q column as previously described [34]. The wild-type Spx protein was overproduced from pMMN470 [31] in ER2566 and purified using a chitin column and a BioRad High S column. To overproduce the Spx^R60E^ protein, pMMN712 was constructed as follows. The fragment, amplified by PCR using oligonucleotides oMMN01-135 and oMMN01-137 together with pMMN683 as template, was digested with NcoI and SmaI and then cloned into pTYB4 (New England Biolabs) digested with the same enzymes. ER2566 carrying pSN21 and pMMN712 were used to overproduce the Spx^G52R^ [8] and Spx^R60E^ mutant proteins, respectively, and the proteins were purified similarly to the wild-type Spx.

### In Vitro Transcription

A linear *trxB* template was generated by PCR with oligonucleotides oDY07-32 and oDY07-52. The template is expected to produce a 66-base transcript. One nM of the template and 25 nM of RNAP together with 25 nM σ^A^ were incubated without or with 7.5 nM Spx protein in 62.3 µl of 10 mM Tris-HCl pH 8.0, 50 mM NaCl, 5 mM MgCl_2_, and 50 µg/ml BSA. After 10-min incubation at 37°C, 7.7 µl of nucleotide mixture (700 µM ATP, GTP and CTP, 35 µM UTP, 17.5 µCi α-^32^PUTP) was added to each reaction. After incubation at 37°C for 2, 5, and 10 min, 20 µl of the reaction was withdrawn to mix with 10 µl of stop solution (1 M ammonium acetate, 0.1 mg yeast RNA, and 0.03 M EDTA). The mixture was precipitated with ethanol and resuspended with 5 µl of formamide-dye (0.3% xylene cyanol, 0.3% bromophenol blue, and 12 mM EDTA dissolved in formamide). The samples were heated at 90°C for 2 min and were applied onto an 8% polyacrylamide-urea gel. The gel was dried and autoradiographs were scanned on a Typhoon Trio scanner (GE Healthcare).

### Electrophoretic Mobility Shift Assay (EMSA)

The probe used for EMSA was a fragment extending from −56 to −21 of the *trxB* promoter region, which was generated by annealing complementary oligonucleotides. The 36-mer oMMN08-465 was the template strand and the 35-mer oMMN08-466 was the non-template strand that lacks A (complementary to −56T) at its 3′-end. The two oligonucleotides (5 pmoles each) were mixed in 20 µl of 10 mM Tris-HCl pH 7.9, 50 mM NaCl, 10 mM MgCl_2_ and heated at 90°C for 5 min, then slowly cooled to room temperature. To radiolabel the non-template strand, Klenow fragment and 10 µCi of [α-^32^P]dATP (800Ci/mmol) were added to the annealing reaction to fill-in the 3′ end. After incubation at room temperature for 15 min, unincorporated [α-^32^P]dATP was removed using a nucleotide purification kit (Qiagen). The mutant (G-44A G-33A) *trxB* and *spoVG* probes were generated in a similar manner, except using oligonucleotides oMMN08-473 and oMMN08-474, and oMMN09-477 and oMMN09-478, respectively.

Five µM of Spx and 5 µM of αCTD (unless otherwise stated) were incubated at room temperature for 10 min in 20 µl of 20 mM Tris-HCl pH 7.8, 50 mM NaCl, 5 mM MgCl_2_, 10% glycerol. The radiolabeled probe (2,000 cpm/reaction) was added to the preincubated mixture and further incubated at room temperature for 15 min. The reaction mixture was applied onto a pre-run 6% native polyacrylamide gel and run in TGE buffer (50 mM Tris, 0.38 M glycine, 2 mM EDTA) at 180V. The gel was dried and scanned on a Typhoon Trio variable mode imager.

## Results

### The Wild-Type and Spx(C10S) Structure

**Figure 1:**
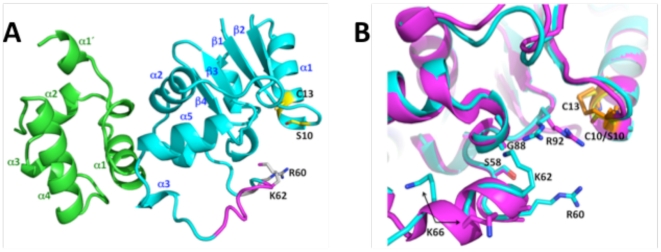
Structure of reduced C10S Spx in complex with αCTD. (A) Spx and αCTD are shown as teal and green ribbons, respectively, and their secondary structures are labelled. Helix α4, which is observed in oxidized Spx [9] but has unraveled in the reduced form, is colored magenta. The residues mutated in this study, R60 and K62, are labelled and shown as sticks with carbon atoms colored white and nitrogen atoms either blue or magenta. Residues S10 and C13 are labelled and shown as sticks with carbon and sulphur atoms colored yellow and the γ-oxygen of S10, red. (B) Close up of the region surrounding helix α4 and residues C10/S10 and C13 after the superposition of the oxidized and reduced αCTD-Spx complex structures. Reduced Spx is shown as a magenta ribbon and oxidized Spx as a teal ribbon. The C10-C13 disulfide bond is shown in orange sticks and S10 and C13 from the reduced structure are shown as yellow sticks. In the reduced form residue R92 has moved 2.8 Å away from its position in the ammonium sulphate-containing oxidized form [9]. The side chain of residue R60 beyond the Cβ atom is disordered in the sulphate-containing crystal form of oxidized Spx, which was used in the superposition visualized here [9].

Formation of the disulfide bond between C10 and C13 is essential for the positive regulatory role of Spx, but not for its negative role [10], [20], [35]. We hypothesized that a conformational change caused by formation of the disulfide bond could provide a mechanism for how Spx is involved in the transcriptional activation of genes such as *trxA* and *trxB*. Therefore, the crystal structure of Spx (C10S), which mimics the reduced form, in complex with the αCTD was determined to 1.9 Å resolution (Figure 1A).

The structure of the reduced αCTD-Spx complex is quite similar to that of the oxidized αCTD-Spx complex [9], [36] and an overlay of 156 corresponding Cα atoms of both complexes, excluding residues on helix α4 of Spx, results in a root mean square deviation of 0.6 A. As seen previously the αCTD contains four core α helices (α1–α4) and a somewhat extended N-terminal helix designated α1′ (Figure 1A). The reduced C10S Spx protein is a mixed α/β protein with its secondary structural elements arranged: β1α1β2α2α3(3_10_)α5β3β4α6 (Figure 1A). Thus, Spx retains most of the secondary structure that is found in oxidized Spx. Importantly, the αCTD-Spx interface of the reduced complex is identical to that of the oxidized αCTD-Spx complex indicating that the biological effects of thiol stress readout by these proteins are not a consequence of a radically different structure of this complex.

Interestingly, the loss of the disulfide linkage between residues C10 and C13 does not result in a significant change in the local structure (Figure 1B). Although free to rotate from their positions in the oxidized state, the S10 and C13 side chains do not move because their positions are buttressed by numerous interactions. Indeed the S10 Oγ side chain engages in hydrogen bonds to the backbone amide (NH) group of C13 and Oγ atom of residue S12. The C13 Sγ sulfhydryl group hydrogen bonds to hydroxyl side chain of residue S10 and makes van der Waals contacts with residues R92 and P93. Also, due to two alternative backbone conformations around residues S7 and P8, the S7 backbone carbonyl oxygen can engage in a weak hydrogen bond to the C13 SHγ group.

One significant conformational difference is found between oxidized and reduced Spx, however, whereby helix α4 of the reduced mutant Spx structure (residues S61 to N68 in oxidized Spx) unfolds and rotates (Figure 1). As a consequence of the unravelling of helix α4, several basic residues are repositioned. Specifically, the side chain of residues K62, which points into the solvent in oxidized Spx and is disordered, turns inward and now makes hydrogen bonds to the carbonyl oxygen (CO) atom of residue G88 and the Oγ atom of residue Ser58. Residue K66, which is also solvent exposed in oxidized Spx, is now pointing into the core of the protein. Hence, the exposed electropositive surface of α4 is decreased significantly in reduced Spx. Other helix α4-related conformational changes include the repositioning of residue R60, whereby its Cα carbon has moved 1.3 Å from its oxidized position. The side chain of the R60 residue, which points directly into the solvent and is disordered in one crystal structure of oxidized Spx but not in the second [36], has moved 5.8 Å (Cζ–Cζ) from its position in the oxidized protein and has shifted towards R92 by 1.3 Å (Figure 1B). The potential importance of this altered location is tied to residue R92. In reduced mutant Spx, the side chain of residue R92 has rotated outward by ∼2.8 Å from its location in oxidized Spx. In the oxidized protein, the guanidinium side chain is engaged in an electrostatic interaction with a bound sulfate ion [9]. In the reduced protein, the R92 side chain rotates inward and makes hydrogen bonds to the peptide backbone carbonyl oxygen atoms of residues G88 and L90 (Figure 1B). This location of the R92 side chain of reduced Spx is very similar to its location in oxidized Spx crystallized from solutions that do not contain sulfate or phosphate anions [36]. Perhaps, the R92-bound sulfate ion described in the first reported oxidized Spx structure is a surrogate for one of the phosphate groups of (αCTD-Spx)-bound DNA. If so, this also places the solvent exposed guanidinium group of residue R60 near the DNA phosphate backbone and suggests its possible role in DNA binding, either to the backbone or to a guanine. Thus, loss of the Spx C10-C13 disulfide bond results only in small conformational changes that are confined primarily to helix α4. However, the resulting helix-to-coil transition repositions the side chains of several basic residues that could have functional consequences with respect to DNA binding.

### Residues in or Near Helix α4 of Spx Are Important for *trxB* Activation but Not for *srfA* Repression by ComA Activator Interference

**Figure 2:**
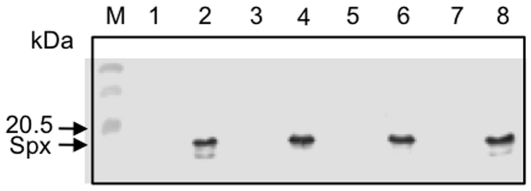
Production of the wild-type and mutant Spx in *B. subtilis*. *B. subtilis* cells expressing the wild-type and the mutant Spx^DD^ (the C-terminal two amino acids are substituted with aspartate residues, which renders the Spx protein insensitive to ClpXP protease) were grown in DS medium in the absence (lanes 1, 3, 5, and 7) and the presence of IPTG (lanes 2, 4, 6, and 8) as described in Materials and Methods. The lysate was prepared by the protoplast lysis method as described and 15 µg of total protein was resolved by SDS-polyacrylamide gel electrophoresis. Western blot analysis was carried out to detect Spx as shown previously. Lanes: M, molecular weight marker; 1 and 2, ORB6894 (Spx^DD^), ORB6895 (Spx^DD-R60E^), ORB6896 (Spx^DD-K62E^), and ORB6897 (Spx^DD-K66E^).

**Figure 3:**
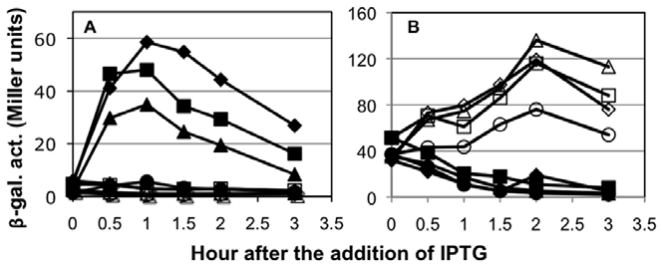
Effect of amino acid substitutions within and near helix α4 of Spx on the transcription of *trxB* (A) and *srfA* (B). Strains carrying *trxB-lacZ* (A) or *srfA-lacZ* (B) were grown in DS medium. When the OD_600_ was 0.4 to 0.5, each culture was divided into two flasks, and 1 mM IPTG was added to one flask to induce Spx^DD^. Samples were taken at time intervals and β-galactosidase activities were measured. (A) Symbols: squares, ORB6894 with Spx^DD^; circles, ORB6895 with Spx^DD-R60E^; triangles, ORB6896 with Spx^DD-K62E^; diamonds, ORB 6897 with Spx^DD-K66E^. (B) Symbols: squares, ORB6129 with Spx^DD^; circles, ORB6934 with Spx^DD-R60E^; triangles, ORB6935 with Spx^DD-K62E^; diamonds, ORB6936 with Spx^DD-K66E^. Open symbols represent cells cultured without IPTG and closed symbols represent cells cultured with IPTG.

To determine whether the structural change in helix α4 is crucial for the function of Spx in transcription activation, we next introduced single amino acid substitutions around the helix α4 region. Interestingly, there are some basic residues adjacent to and within the helix, namely, R60, K62, and K66. Since basic amino acids are known to interact with DNA through sequence recognition and charge neutralization [37], we decided to substitute each residue with glutamate and to examine the effect of these substitutions on Spx activity. Because under nonstress conditions Spx is degraded by ClpXP protease, we expressed the ClpXP-resistant forms (Spx^DD^) of the wild-type and mutant proteins from an IPTG-inducible promoter, as previously described [2] so that we could examine the mutational effect on *trxB* transcription under nonstress conditions. Western blot analysis showed that the three mutant Spx proteins were produced only in the presence of IPTG at a level similar to the wild-type protein (Figure 2). *trxB-lacZ* expression in cells producing Spx^K66E^ was equal to or greater than that observed in cells producing the wild-type Spx (Figure 3A). In contrast, the K62E mutation reduced transcription and the R60E mutation nearly abolished transcription, indicating that R60, and to a lesser extent K62, are important for transcriptional activation of *trxB*.

Although Spx^R60E^ and Spx^K62E^ are produced at a level similar to the wild-type Spx in *B. subtilis* cells, we could not completely eliminate the possibility that the mutant proteins were misfolded, and thus, inactive. As described earlier, Spx plays both a positive and negative role in transcription regulation. To examine whether the mutant proteins retain the ability to exert negative transcriptional control, we determined the effect of the mutations on ComA-dependent *srfA* transcription. As shown in Figure 3B, *srfA* transcription was severely reduced in a strain that produced the wild-type Spx protein and in all of the strains that produced the mutant proteins. These results clearly demonstrated that R60 and K62 of Spx play pivotal roles in positive control but are dispensable for its negative role in transcription.

### Identification of *trxB* Sequences Required for Spx-Dependent Transcription Activation

One possible hypothesis for why R60 (and to a lesser extent K62) is required for *trxB* transcription and not for inhibition of activator-dependent *srfA* transcription is that R60 is involved in the interaction of Spx with the *trxB* promoter DNA for establishing the transcription initiation complex. Although Spx itself did not bind *trxB* DNA [10], this result does not necessarily eliminate the possibility that Spx, by interacting with αCTD, can contribute part of a DNA-binding surface. The intracellular disulfide bond formation might facilitate the recognition and/or binding of the side-chain of R60 with a specific nucleotide in the *trxB* promoter region, as suggested above. Alternatively, Spx residue R60 might function indirectly in DNA sequence recognition by changing the conformation of αCTD so that it recognizes specific sequences associated with Spx-activated promoters. We think that this is unlikely as explained in the Discussion.

Our previous work demonstrated that the *trxB* promoter region between −50 and −36 is required for Spx-dependent transcription activation and that the nucleotides at positions −43 and −44 are essential for transcription [10], [11]. The identified region corresponds well with the sequence protected from DNase I digestion in the presence of the wild-type Spx-RNAP complex [10]. The DNase I footprinting analyses also identified a hypersensitive site between −34 and −35 of the *trxB* template strand in the presence of Spx-RNAP [10]. Interestingly, Spx-RNAP generated a hypersensitive site at a similar position (between −35 and −36) of *trxA*, a gene that is also activated directly by Spx. Furthermore, the nucleotide sequences (AAAATAGCGT) of the *trxA* (−40 to −31) and *trxB* (−39 to −30) regions that include the hypersensitive site are identical. These observations prompted us to carry out further mutational analyses of the −39 to −30 region of *trxB*.

**Figure 4:**
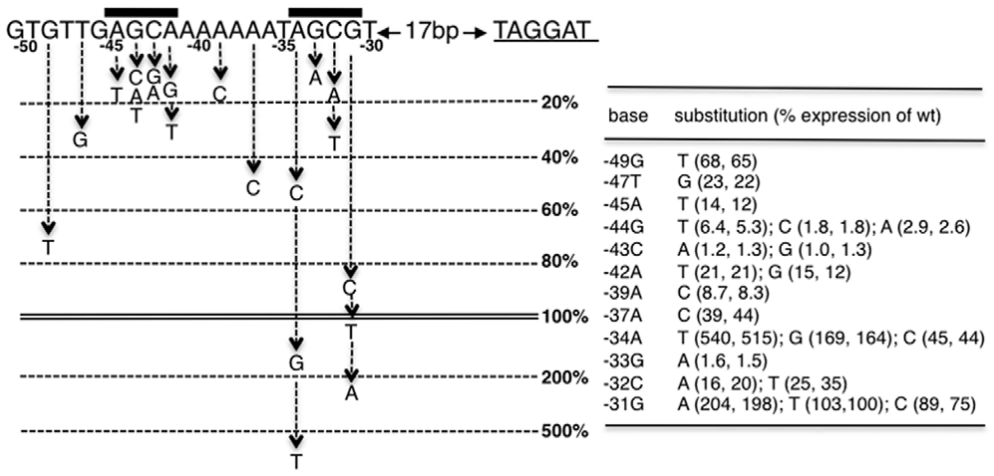
Effect of base substitutions of the *trxB* promoter on *trxB* expression. Single base pair substitutions were generated in the *trxB* promoter (−115 to +47). The mutated promoters fused to *lacZ* were introduced in *spx* mutant strains expressing *spx^DD^* from the IPTG-inducible P*spank-hy* promoter. Expression of *trxB-lacZ* was determined in at least two independent isolates as described in Fig. 3. The effect of each base substitution is shown as a percentage of the peak *trxB* transcribed from the wild-type promoter, which was used as a control in each experiment. The peak expression was generally seen around 1.5 hr after the addition of IPTG.

As in the previous study [11], we used the *trxB* promoter carrying −115 to +47 for base substitution experiments, except that we expressed the IPTG-inducible Spx^DD^ in the strain lacking the native *spx* gene. The results showed that three segments of *trxB* are important for Spx-dependent transcription activation (Figure 4). The first segment is the AGCA sequence positioned from −45 to −42. Our previous study showed that the G at −44 and C at −43 are indispensable for transcription [10], [11]. The second segment is a poly-A stretch between −41 and −36. In our previous study, base substitution of two of the A residues showed a moderate effect on *trxB* transcription in the *spx* ^+^ background [11], and here, these substitutions showed more adverse effects in cells lacking the native *spx* gene (Figure 4). It has been known that αCTD binds to AT-rich sequences; for example, the sequence AAAAAARNR at positions −46 to −38 of the *E. coli rrnB* promoter P1 serves as the proximal αCTD-binding site [38]. We, therefore, propose that the sequence between −41 and −35 is a site for αCTD interaction. The last segment important for *trxB* transcriptional control by Spx is the GC sequence positioned at −33 and −32. Our previous work showed that Spx-dependent activation of a *trxB/srfA* hybrid promoter transcription was enhanced further by extending the *trxB* control region to −31 including the GC at −32/33 [11]. The dinucleotide resides in the center of the AGCG sequence that is similar to the upstream essential AGCA (−45 to −42) sequence. The G residue in the second position is the most critical among the tetranucleotides AGCA and AGCG. Because the two tetranucleotides are similar, with the exception that the most 3′-end of the upstream sequence is A (−42) the corresponding position of the downstream sequence is G (−31), we examined the effect of base substitution of the G at position −31. We found that substitution of G with either C or T had no significant effect, whereas the substitution with A led to a more than two-fold increase in *trxB* expression. One interpretation of this result is that the Spx/αCTD complex binds to *trxB* and that Spx contacts the AGCA sequence and αCTD contacts the downstream A-rich sequence. If this is true, then the question is which protein, if any, binds to the downstream AGCG sequence. The previous crosslinking study did not show contact of any protein to the −35 region [11], and was, therefore, inconclusive. One could envision three alternative scenarios for a protein/−35 interaction. One scenario is that σ^A^ binds to this sequence, which is facilitated by the interaction between σ^ A^ and αCTD bound to the site upstream of −35. Another scenario is that the downstream AGCG sequence is a site where Spx binds. A third possibility is that the second αCTD, having undergone a conformational change upon contact with oxidized Spx, now recognizes the sequence centered at −33. For the reasons presented in the Discussion, this possibility seems unlikely. The issue of −33 element recognition will be discussed below (see Discussion).

### Activation-Defective Spx Mutants Other Than G52R Are Able to Activate a Mutant *trxB* Promoter Bearing an A-34T Mutation

**Figure 5:**
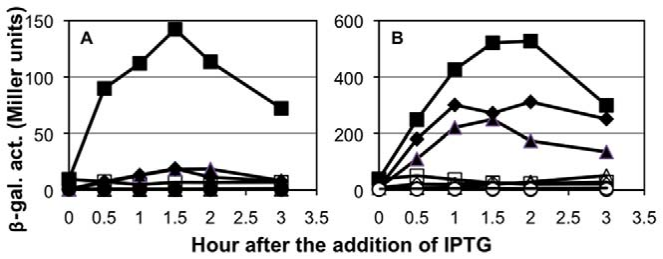
Effect of the A-34T substitution on *trxB* transcription activated by the wild-type and mutant Spx. Strains carrying *trxB-lacZ* fusions were grown in DS medium. When the OD_600_ was 0.4 to 0.5, each culture was divided into two flasks, and 1 mM IPTG was added to one flask. Samples were taken at time intervals and β-galactosidase activities were measured. (A) Expression of the wild-type *trxB-lacZ*. Symbols: squares, ORB7276 with Spx^DD^; triangles, ORB7282 with Spx^DD-R60E^; diamonds, ORB7316 with Spx^DD-C10A^; circles, ORB7337 with Spx^DD-G52R^. (B) Expression of *trxB*(A-34T)-*lacZ*. Symbols: squares, ORB7342 with Spx^DD^; triangles, ORB7343 with Spx^DD-R60E^; diamonds, ORB7347 with Spx^DD-C10A^; circles, ORB7348 with Spx^DD-G52R^. Open symbols represent cells cultured without IPTG and closed symbols represent cells cultured with IPTG.

The mutational analysis showed that the A-34T substitution resulted in highly elevated *trxB* transcription, which remained largely dependent on Spx because IPTG was still required for promoter activity (Figure 4). We examined expression of *trxB*(A-34T)-*lacZ* in cells expressing *spx^R60E^*, *spx^C10A^*, and *spx^G52R^* mutants, as well as the wild-type *spx*. As shown in Figure 5A, transcription from the wild-type *trxB* promoter was severely reduced in cells producing each mutant Spx^DD^ protein as compared with those producing wild-type Spx^DD^. In contrast, the R60E and C10A Spx mutants were able to activate the A-34T promoter, although the activity of the mutant proteins was approximately 50% of the activity of the wild-type protein (Figure 5B). No expression was observed in the absence of IPTG, indicating that the observed expression is dependent on mutant Spx protein. Unlike the two Spx activation mutants, the Spx^G52R^ mutant protein was completely impaired in activating transcription from the mutant promoter. This result indicates that the G52 residue, and hence, the Spx-αCTD interaction is absolutely required for Spx-activated *trxB* transcription, but the requirement of residues R60 and C10 is conditional, given that the *spx* mutations R60E and C10A are partially suppressed by the A-34T mutation in the *trxB* promoter.

### Spx^R60E^ Activates *trxB*(A-34T) Transcription *In Vitro*

**Figure 6:**
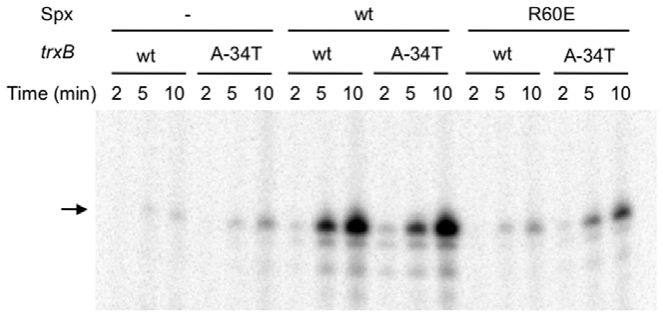
In vitro transcription from the wild-type and *trxB*(A-34T) promoters in the absence and presence of the wild-type and R60E Spx. Either the wild-type or A-34T *trxB* template (1 nM) was incubated with 25 nM RNAP together with 25 nM σ^A^ in the presence of 7.5 nM Spx. The arrow shows the 66-base *trxB* transcript.

The *in vivo* results described above showed that Spx^R60E^ and Spx^C10A^ are unable to activate transcription from the wild-type *trxB* promoter, but are able to significantly activate *trxB*(A-34T) transcription. We next carried out *in vitro* run-off transcription experiments to determine whether the effect of the A-34T substitution can be solely attributed to interactions involving the *trxB*(A-34T) promoter, Spx, and RNAP. Figure 6 shows that the basal level of *trxB* transcription was markedly elevated by adding wild-type Spx, but only a slight increase in the transcript was detected in reactions containing Spx^R60E^. The *trxB*(A-34T) transcript accumulated slightly more than the wild-type *trxB* transcript at a longer reaction time (10 min) in the absence of Spx, and the transcript levels were increased further when the wild-type Spx was present. Unlike the wild-type transcript levels, those of the mutant *trxB* were markedly elevated by Spx^R60E^. We repeated these experiments almost ten times and in some experiments we did not see any difference between the wild-type and the mutant *trxB* transcript levels activated by the wild-type Spx as shown in Figure 6; however, in other experiments, we observed that the mutant *trxB* transcript level was significantly higher than the wild-type transcript level. Although we do not understand the variability in the in vitro transcription results, Spx^R60E^ reproducibly activated transcription from the t*rxB*(A-34T) promoter to a higher level than from the wild-type promoter. Based on the *in vitro* transcription assays, we conclude that the adverse effect of the R60E mutation on *trxB* transcription is likely caused by either a weaker interaction of the mutant Spx with the *trxB* promoter, RNAP, or both.

### The Spx-αCTD Complex Binds the *trxB* Regulatory Region

**Figure 7:**
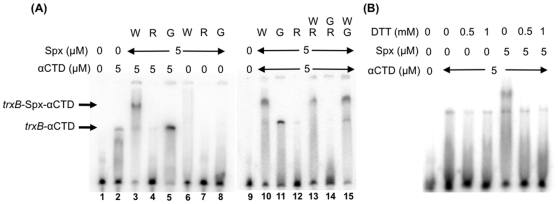
Interaction of αCTD and Spx variants with the regulatory region of the *trxB* promoter. The *trxB* probe (−56 to −21) was generated by annealing of oligonucleotides followed by labeling of the 3′-end of the template strand using Klenow fragment and [^32^P]dATP. Bands corresponding to the *trxB*/αCTD and *trxB*/Spx/αCTD complexes are marked with arrows. (A) EMSA analysis of αCTD and Spx binding to the *trxB* probe in reactions containing Spx variant or mixtures of mutant Spx proteins or mutant with the wild-type Spx (each at 5 µM). Abbreviations: W, wild-type Spx; G, Spx^G52R^; R, Spx^R60E^. (B) Redox-sensitive interaction was examined in the presence of DTT.

The mutational studies of the *trxB* promoter uncovered a region of *trxB* required for Spx-dependent activation [10], [11], and this putative *cis*-acting component of Spx control was further investigated. Given that the A-rich sequence and the flanking AGCA and AGCG sequences are conserved between the *trxA* and *trxB* promoters and that the upstream AGCA sequence is also present in other Spx-controlled genes (see Discussion), one could envisage that the AGCA sequence (and possibly AGCG in *trxA* and *trxB*) is the site where Spx binds. To test this possibility, we next carried out EMSA analysis using a DNA fragment carrying the *trxB* promoter (−56 to −21). This fragment covers the putative Spx- and αCTD-binding sites, but lacks the −10 region. We first examined whether either αCTD or Spx alone binds DNA. αCTD at 5 µM bound DNA, but Spx at the same concentration did not (Figure 7A, lanes 2 and 6). Although Spx itself was unable to bind DNA at the concentration tested, Spx addition resulted in a supershifted complex with promoter DNA and αCTD, indicating that a DNA-αCTD-Spx ternary complex was formed (Figure 7A, lane 3). In contrast, the supershifted band was not detected with Spx^G52R^ (Figure 7A, lane 5), arguing that formation of the ternary complex is dependent on the interaction of Spx and αCTD. The effect of R60E Spx on the DNA-αCTD complex was completely different from that of either the wild-type Spx or Spx^G52R^; Spx^R60E^ prevented DNA and αCTD from forming a complex (Figure 7A, lane 4). Furthermore, addition of Spx^G52R^ did not affect the ternary complex formed by the wild-type Spx (Figure 7A, lane 15). In contrast, addition of Spx^R60E^ negatively affected the DNA-αCTD-wild-type Spx complex (Figure 7A, lane 13), and completely abolished the DNA-αCTD binary complex (Figure 7A, lane 14) when it was included in the binding reaction with the G52R protein (compare with Figure 7A, lane 11, a reaction containing the G52R mutant protein alone with αCTD). The ternary complex, but not the binary complex, was only formed in the absence of DTT (Figure 7B), which is in good agreement with the hypothesis that the oxidized form of Spx is required for DNA binding.

**Figure 8:**
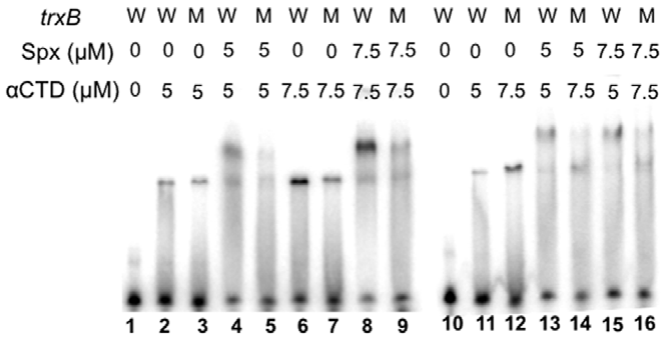
Effect of *trxB* base substitutions on interaction with αCTD and Spx. The wild-type (W) and the *trxB* (G-44A/G-33A) mutant (M) probes were incubated with different concentrations of αCTD and Spx as described in Figure 7.

We next examined whether the *trxB* mutations that affect Spx-dependent activation had lower binding affinities for the Spx-αCTD complex. The *trxB* promoter fragment bearing the G-44A and G-33A mutations was used for a probe in EMSA to compare the binding affinity for αCTD and Spx-αCTD (Figure 8). When Spx was added in equal concentrations in reactions containing *trxB*(wt)-αCTD or *trxB*(G-44A G-33A)-αCTD, the ternary complex was more abundant when the wild-type promoter was present than when the mutant promoter was present (Figure 8, lanes 4 and 5, and 8 and 9). Even in the reaction in which more αCTD bound to the mutant promoter than the wild-type promoter, Spx was unable to supershift the mutant promoter complex as efficiently as the wild-type promoter complex (Figure 8, lanes 11 to 16). A mutant *trxB-lacZ* fusion bearing the two nucleotide substitutions produced 0.7 units of β-galactosidase activity (data not shown). These results support the assumption that G-44A (and G-33A) is important for the binding of Spx to the *trxB* promoter. We carried out a similar experiment with the *trxB* promoter carrying either the G-44A or G-33A mutation to determine which residue is critical for Spx binding; however, the *trxB* promoter carrying the single mutation did not show a significant difference from the wild-type promoter in EMSA analysis (data not shown).

**Figure 9:**
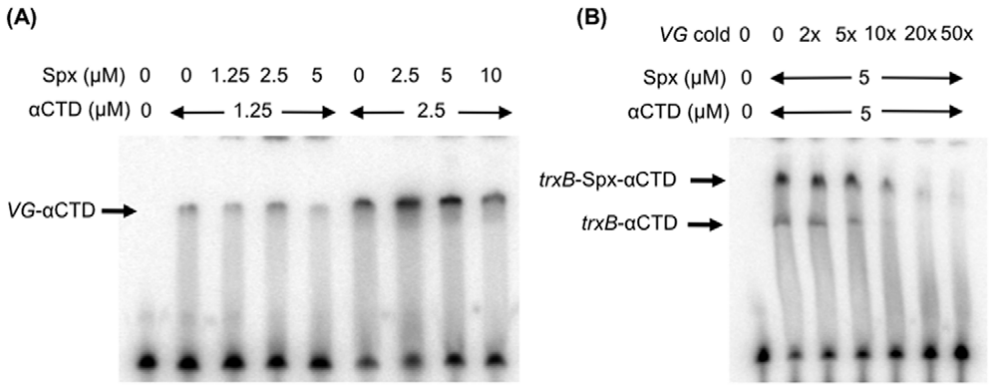
Interaction of αCTD and Spx with the *spoVG* promoter carrying AT-rich upstream sequences. (A) The radiolabeled *spoVG* probe was generated as described in Figure 7. The *spoVG*-αCTD complex is marked with an arrow. (B) Competition of the *trxB*-αCTD and *trxB*-Spx-αCTD complexes with *spoVG* was examined by the addition of a 2- to 50-fold excess of cold *spoVG* probes.

We examined whether the DNA-αCTD-Spx complex is specific to promoters of Spx-activated genes by using the *spoVG* promoter in a similar EMSA analysis. The *spoVG* promoter has an AT-rich upstream sequence that was shown to have properties of an UP element, and was required for transcription [39], [40]. Spx did not activate transcription of *spoVG*; conversely, the transcription was shown to be inhibited by overproduction of Spx through an as yet undiscovered mechanism [41], [42]. The *spoVG* promoter exhibited a much higher affinity for αCTD than the *trxB* promoter, yet addition of Spx did not result in a supershifted DNA-αCTD complex even when an excess of Spx over αCTD was added (Figure 9A). In addition, when the cold *spoVG* fragment was added, it was able to disrupt both αCTD- and αCTD-Spx-binding to the *trxB* promoter (Figure 9B). These results suggest that *spoVG* has a higher affinity for free αCTD than αCTD complexed with Spx, and once αCTD binds the *spoVG* promoter, Spx is unable to establish an interaction with αCTD.

## Discussion

This study was aimed at elucidating how the oxidized form of Spx activates *trxB* transcription. The questions to be answered were: 1) how disulfide-bond formation at the redox CXXC center of Spx affects its activity as a transcriptional activator; 2) whether oxidized Spx in the Spx-αCTD complex binds to the *trxB* promoter region and enhances the binding of αCTD or other subunits of RNAP to DNA; 3) if Spx binds to DNA, what is the consensus Spx-binding site in *trxB* and other Spx-activated promoters. A comparison of the crystal structures of the oxidized (wild-type) and reduced (C10S) forms of Spx revealed that in the reduced form helix α4 partially unfolds and rotates, suggesting that helix α4 could be important for the positive role of Spx in transcription. Consistent with this assumption, mutations of Spx residues R60 and K62, which are adjacent to and within helix α4, respectively, reduced Spx-dependent *trxB* transcription but did not show any effect on its repression of ComA-dependent *srfA* expression. We found that the R60 and K62 residues are well conserved among Spx orthologs, whereas residue 66 is a glutamate instead of lysine in Spx from some *Bacillus* species such as *Bacillus cereus*, *Bacillus thuringiensis*, *Bacillus clausii*, *Bacillus halodurans*, and *Bacillus weihenstephanensis*. The observation supports the important role of residues R60 and K62 in the function of Spx as a transcriptional activator.

The current mutational analysis of the *trxB* promoter not only confirmed the results from our previous work, but also further defined the *cis*-sequences required for Spx-dependent activation. Based on the mutational analyses and the EMSA experiments, we now propose that the AGCA sequence at −45 to −42 is the site with which the complex of αCTD with oxidized Spx directly interacts. This hypothesis is further supported by the following observations. Our previous microarray analysis [2] identified *nfrA*, coding for nitro/flavin reductase [43], [44], as one of the genes activated by Spx. *nfrA* was also shown to be activated in response to a number of stress condition, including oxidative stress [43], [45] and the *nfrA* promoter contains the AGCA sequence at the same position (−45 to −42) as in the *trxB* promoter. Base substitutions of the first three nucleotides in this sequence, particularly G and C, resulted in a substantial reduction in the promoter activity, indicating the essential role of the AGCA sequence in *nfrA* transcription [43]. Our studies further showed that Spx activates *nfrA-lacZ* in vivo and the R60E mutation in Spx severely affects *nfrA* expression (A. L, and P. Z., unpublished results). In addition, an *in vitro* transcription assay showed that Spx directly activates *nfrA* transcription (A.L. and P.Z., unpublished results). The results are in good agreement with the hypothesis that the helix α4 of Spx functions in the interaction of αCTD/Spx complex with the AGCA sequence. We feel that it is unlikely that a conformational change of αCTD caused by Spx is responsible for the recognition of promoter DNA solely by the α polypeptide, as no uncharacteristic change in a conformation is observed when αCTD is bound to Spx [9], [36].

In contrast to the AGCA sequence, the downstream AGCG sequence at −34 to −31, which is conserved in *trxA* and *trxB*, is absent in the *nfrA* promoter; hence, its role in transcriptional activation is unclear. Interestingly, the substitution of A at −34 with T resulted in a slightly higher basal level of *trxB* expression and a five-fold increase in Spx-dependent activation as compared with transcription from the wild-type promoter (Figures 4 and 5). Furthermore, *trxB* transcription from the mutant promoter was significantly stimulated by Spx^R60E^ and Spx^C10A^ (Figures 5 and 6), whereas the G52 residue was absolutely required for transcription. The A-34T change leads to a 3/6 match to the consensus −35 hexamer recognized by σ^A^RNAP [46]. One possible scenario is that σ^A^ binds to the −35 region of the mutant promoter and the interaction between Spx and αCTD at the −44 element, as well as interaction of αCTD with σ^A^, stabilizes the three proteins at the mutant promoter, which partially compensates for the defect in *trxB* interaction conferred by the R60E substitution. In contrast, the G52R mutation, by disrupting the interaction of Spx with αCTD, destabilizes αCTD binding to *trxB*, resulting in decreased engagement of σ^A^ with the −35 region. Does this possibility suggest that σ^A^ also interacts with the −35 region of the wild-type *trxB* promoter? Another mutation, C at −32 to A, which results in a 3/6 match to the consensus −35 hexamer did not increase *trxB* transcription, and instead, showed a severe adverse effect, suggesting that σ^A^ does not bind to the −35 of region the wild-type promoter and/or that the A-34T mutation increases the affinity of σ^A^ binding more than C-32A. The A-34T change leads to the sequence TTGCGT. The TTG in the −35 region seem to be the most important nucleotides of the −35 hexamer as these are the most highly conserved [46], which could explain the opposite phenotypes conferred by the C-32A and A-34T mutations.

Our previous study [11] showed that σ^A^ crosslinks to *trxB* at position −11 but not at −34. Furthermore, Spx did not crosslink to any nucleotide tested. One possible reason for this negative result may be the limited positions tested in the crosslinking experiments. Within the three important regions found in this work, the nucleotide positions tested for crosslinking were −46 and −34 of *trxB* and −47, −35, and −30 of *trxA*. Only −34A of *trxB* and −35A of *trxA* reside within the AGCG sequence. Another possible reason for the negative result is that the modification of a nucleotide with azidophenacyl bromide might have interfered with the binding of either Spx or σ^A^ to DNA. It would be worth revisiting the nucleotide-specific crosslinking study by focusing on the AGCA and AGCG sites, as well as the A-rich sequence, and by confirming that the modified templates are transcriptionally active.

Direct interaction between protein and DNA can also be verified genetically by site-specific suppressor analysis. Earlier studies of the sigma subunit-DNA interaction [47], [48] demonstrated that mutations of the third G of the −35 hexamer to either A or C, but not to T, were suppressed by the substitution of Arg588 in region 4 of σ^70^ with His. Similarly, the defect caused by substitution of the fifth C of the −35 hexamer with either T or G was compensated for by the substitution of Arg584 of σ^70^ with Cys or His. We have investigated whether the various single base substitutions of G at −44, C at −43, G at−33, or C at −32, as well as double mutation of either G at −44/−33 or C at −43/−32, were restored by introducing the R60H or R60C mutations in Spx; however, we could not detect any significant suppressing effect in any of the mutant combinations (M.M.N. and P.Z. unpublished results).

The αCTD of RNAP can make specific contact with the UP element sequence of certain promoters such as those of rRNA operons [49] and the *spoVG* gene of *B. subtilis* (Figure 9). Evidently, the αCTD in the Spx-αCTD complex engages the *trxB* promoter DNA in a manner that is different from its interaction with the UP element, as Spx is unable to contact αCTD on the *spoVG* promoter fragment. Some of the αCTD residues required for UP element contact might also function in its interaction with Spx, and thus, may not be available for Spx-αCTD complex formation. The α1 helix of αCTD contains part of the “261 element” that is required for DNA binding, and this helix also contains the essential Tyr residue for Spx interaction. Given that part of the α1 helix interacts with Spx further suggests that only part of the DNA-binding surface in αCTD might be exposed for DNA recognition, and that interaction with Spx is required to complete the DNA binding surface of the αCTD/Spx promoter recognition complex.

We, and others, have successfully cocrystallized αCTD and oxidized Spx as previously reported [9], [36]. The EMSA study presented here now opens a powerful approach for cocrystallization of the αCTD-Spx complex with DNA to study the ternary interaction.

## References

[1] Zuber P (2004). Spx-RNA polymerase interaction and global transcriptional control during oxidative stress.. J Bacteriol.

[2] Nakano S, Küster-Schöck E, Grossman AD, Zuber P (2003). Spx-dependent global transcriptional control is induced by thiol-specific oxidative stress in *Bacillus subtilis*.. Proc Natl Acad Sci USA.

[3] Bergman NH, Passalacqua KD, Hanna PC, Qin ZS (2007). Operon prediction for sequenced bacterial genomes without experimental information.. Appl Environ Microbiol.

[4] Chatterjee SS, Hossain H, Otten S, Kuenne C, Kuchmina K (2006). Intracellular gene expression profile of *Listeria monocytogenes*.. Infect Immun.

[5] Hochgrafe F, Wolf C, Fuchs S, Liebeke M, Lalk M (2008). Nitric oxide stress induces different responses but mediates comparable protein thiol protection in *Bacillus subtilis* and *Staphylococcus aureus*.. J Bacteriol.

[6] Kajfasz JK, Martinez AR, Rivera-Ramos I, Abranches J, Koo H (2009). Role of Clp proteins in expression of virulence properties of *Streptococcus mutans*.. J Bacteriol.

[7] Pamp SJ, Frees D, Engelmann S, Hecker M, Ingmer H (2006). Spx is a global effector impacting stress tolerance and biofilm formation in *Staphylococcus aureus*.. J Bacteriol.

[8] Nakano S, Nakano MM, Zhang Y, Leelakriangsak M, Zuber P (2003). A regulatory protein that interferes with activator-stimulated transcription in bacteria.. Proc Natl Acad Sci USA.

[9] Newberry KJ, Nakano S, Zuber P, Brennan RG (2005). Crystal structure of the *Bacillus subtilis* anti-alpha, global transcriptional regulator, Spx, in complex with the alpha C-terminal domain of RNA polymerase.. Proc Natl Acad Sci USA.

[10] Nakano S, Erwin KN, Ralle M, Zuber P (2005). Redox-sensitive transcriptional control by a thiol/disulphide switch in the global regulator, Spx.. Mol Microbiol.

[11] Reyes DY, Zuber P (2008). Activation of transcription initiation by Spx: formation of transcription complex and identification of a *cis*-acting element required for transcriptional activation.. Mol Microbiol.

[12] Leelakriangsak M, Kobayashi K, Zuber P (2007). Dual negative control of *spx* transcription initiation from the P3 promoter by repressors PerR and YodB in *Bacillus subtilis*.. J Bacteriol.

[13] Leelakriangsak M, Zuber P (2007). Transcription from the P3 promoter of the *Bacillus subtilis spx* gene is induced in response to disulfide stress.. J Bacteriol.

[14] Nakano S, Zheng G, Nakano MM, Zuber P (2002). Multiple pathways of Spx (YjbD) proteolysis in *Bacillus subtilis*.. J Bacteriol.

[15] Garg SK, Kommineni S, Henslee L, Zhang Y, Zuber P (2009). The YjbH protein of *Bacillus subtilis* enhances ClpXP-catalyzed proteolysis of Spx.. J Bacteriol.

[16] Larsson JT, Rogstam A, von Wachenfeldt C (2007). YjbH is a novel negative effector of the disulphide stress regulator, Spx, in *Bacillus subtilis*.. Mol Microbiol.

[17] Zhang Y, Zuber P (2007). Requirement of the zinc-binding domain of ClpX for Spx proteolysis in *Bacillus subtilis* and effects of disulfide stress on ClpXP activity.. J Bacteriol.

[18] Nakano MM, Xia L, Zuber P (1991). Transcription initiation region of the *srfA* operon which is controlled by the *comP-comA* signal transduction system in *Bacillus subtilis*.. J Bacteriol.

[19] Roggiani M, Dubnau D (1993). ComA, a phosphorylated response regulator protein of *Bacillus subtilis*, binds to the promoter region of *srfA*.. J Bacteriol.

[20] Zhang Y, Nakano S, Choi SY, Zuber P (2006). Mutational analysis of the *Bacillus subtilis* RNA polymerase alpha C-terminal domain supports the interference model of Spx-dependent repression.. J Bacteriol.

[21] Geng H, Zuber P, Nakano MM (2007). Regulation of respiratory genes by ResD-ResE signal transduction system in *Bacillus subtilis*.. Methods Enzymol.

[22] Leslie AGW (1992). Recent changes to the MOSFLM package for processing film and image plate data.

[23] Jones TA, Zou JY, Cowan SW, Kjeldgaard M (1991). Improved methods for building protein models in electron density maps and the location of errors in these models.. Acta Crystallogr A.

[24] Brunger AT, Adams PD, Clore GM, DeLano WL, Gros P (1998). Crystallography & NMR system: A new software suite for macromolecular structure determination.. Acta Crystallogr D Biol Crystallogr.

[25] Laskowski RA, Moss DS, Thornton JM (1993). Main-chain bond lengths and bond angles in protein structures.. J Mol Biol.

[26] Britton RA, Eichenberger P, Gonzalez-Pastor JE, Fawcett P, Monson R (2002). Genome-wide analysis of the stationary-phase sigma factor (sigma-H) regulon of *Bacillus subtilis*.. J Bacteriol.

[27] Dahl MK, Meinhof CG (1994). A series of integrative plasmids fo*r Bacillus subtilis* containing unique cloning sites in all three open reading frames for translational *lacZ* fusions.. Gene.

[28] Guerout-Fleury AM, Frandsen N, Stragier P (1996). Plasmids for ectopic integration in *Bacillus subtilis*.. Gene.

[29] Harwood CR, Cutting SM (1990). Molecular Biological Methods for Bacillus..

[30] Miller JH (1972). Experiments in molecular genetics..

[31] Nakano MM, Hajarizadeh F, Zhu Y, Zuber P (2001). Loss-of-function mutations in *yjbD* result in ClpX- and ClpP-independent competence development of *Bacillus subtilis*.. Mol Microbiol.

[32] Liu J, Zuber P (2000). The ClpX protein of *Bacillus subtilis* indirectly influences RNA polymerase holoenzyme composition and directly stimulates sigmaH-dependent transcription.. Mol Microbiol.

[33] Qi Y, Hulett FM (1998). PhoP-P and RNA polymerase sigmaA holoenzyme are sufficient for transcription of Pho regulon promoters in *Bacillus subtilis*: PhoP-P activator sites within the coding region stimulate transcription in vitro.. Mol Microbiol.

[34] Nakano MM, Geng H, Nakano S, Kobayashi K (2006). The nitric oxide-responsive regulator NsrR controls ResDE-dependent gene expression.. J Bacteriol.

[35] Turlan C, Prudhomme M, Fichant G, Martin B, Gutierrez C (2009). SpxA1, a novel transcriptional regulator involved in X-state (competence) development in *Streptococcus pneumoniae*.. Mol Microbiol.

[36] Lamour V, Westblade LF, Campbell EA, Darst SA (2009). Crystal structure of the in vivo-assembled *Bacillus subtilis* Spx/RNA polymerase α subunit C-terminal domain complex.. J Struct Biol.

[37] Benoff B, Yang H, Lawson CL, Parkinson G, Liu J (2002). Structural basis of transcription activation: the CAP-alpha CTD-DNA complex.. Science.

[38] Estrem ST, Ross W, Gaal T, Chen ZW, Niu W (1999). Bacterial promoter architecture: subsite structure of UP elements and interactions with the carboxy-terminal domain of the RNA polymerase alpha subunit.. Genes Dev.

[39] Banner CD, Moran CP, Losick R (1983). Deletion analysis of a complex promoter for a developmentally regulated gene from *Bacillus subtilis*.. J Mol Biol.

[40] Frisby D, Zuber P (1991). Analysis of the upstream activating sequence and the site of carbon/nitrogen source repression in the promoter of an early-induced sporulation gene of *Bacillus subtilis*.. J Bacteriol.

[41] Liu J, Cosby WM, Zuber P (1999). Role of Lon and ClpX in the post-translational regulation of a sigma subunit of RNA polymerase required for cellular differentiation of *Bacillus subtilis*.. Mol Microbiol.

[42] Nakano MM, Zhu Y, Liu J, Reyes DY, Yoshikawa H (2000). Mutations conferring amino acid residue substitutions in the carboxy-terminal domain of RNA polymerase α can suppress *clpX* and *clpP* with respect to developmentally regulated transcription in *Bacillus subtilis*.. Mol Microbiol.

[43] Moch C, Schrogel O, Allmansberger R (2000). Transcription of the *nfrA-ywcH* operon from *Bacillus subtilis* is specifically induced in response to heat.. J Bacteriol.

[44] Zenno S, Kobori T, Tanokura M, Saigo K (1998). Purification and characterization of NfrA1, a *Bacillus subtilis* nitro/flavin reductase capable of interacting with the bacterial luciferase.. Biosci Biotechnol Biochem.

[45] Tam LT, Antelmann H, Eymann C, Albrecht D, Bernhardt J (2006). Proteome signatures for stress and starvation in *Bacillus subtilis* as revealed by a 2-D gel image color coding approach.. Proteomics.

[46] Helmann JD (1995). Compilation and analysis of *Bacillus subtilis* sigma A-dependent promoter sequences: evidence for extended contact between RNA polymerase and upstream promoter DNA.. Nucleic Acids Res.

[47] Gardella T, Moyle H, Susskind MM (1989). A mutant *Escherichia coli* sigma 70 subunit of RNA polymerase with altered promoter specificity.. J Mol Biol.

[48] Siegele DA, Hu JC, Walter WA, Gross CA (1989). Altered promoter recognition by mutant forms of the sigma 70 subunit of *Escherichia coli* RNA polymerase.. J Mol Biol.

[49] Ross W, Ernst A, Gourse RL (2001). Fine structure of *E. coli* RNA polymerase-promoter interactions: alpha subunit binding to the UP element minor groove.. Genes Dev.

